# Measurement of Human and Bovine Exhaled Breath Condensate pH Using Polyaniline-Modified Flexible Inkjet-Printed Nanocarbon Electrodes

**DOI:** 10.1021/acsomega.4c05800

**Published:** 2024-09-16

**Authors:** Aaron I. Jacobs, Maiyara C. Prete, Andreas Lesch, Angel Abuelo Sebio, César Ricardo Teixeira Tarley, Greg M. Swain

**Affiliations:** † Department of Chemistry, Michigan State University, 578 South Shaw Lane, East Lansing, Michigan 48824, United States; ‡ Department of Chemistry, State University of Londrina (UEL), Londrina, Paraná 86051-990, Brazil; § Department of Industrial Chemistry “Toso Montanari”, University of Bologna, Viale del Risorgimento 4, Bologna 40136, Italy; ∥ Department of Large Animal Clinical Sciences, College of Veterinary Medicine, Michigan State University, 736 Wilson Road, East Lansing, Michigan 48824, United States

## Abstract

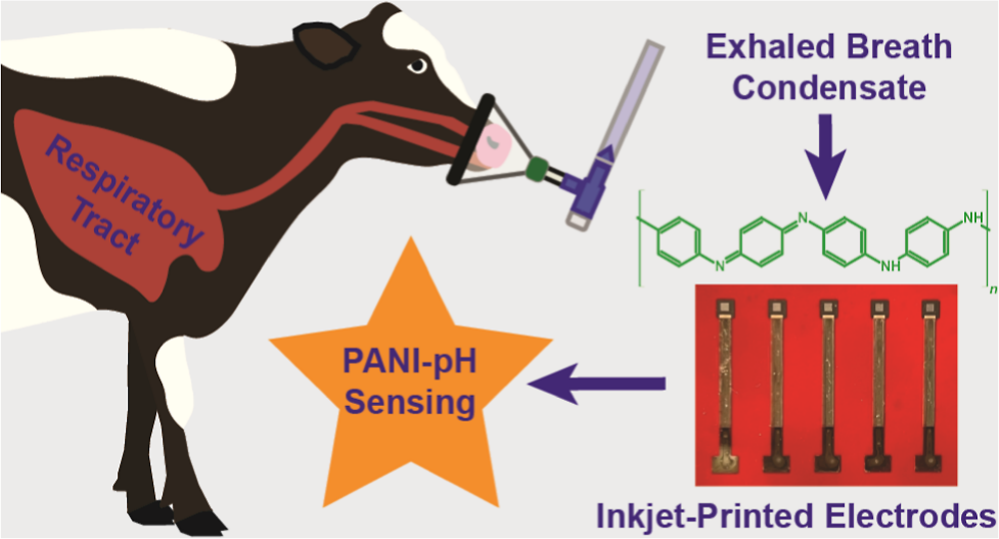

The collection, processing, and electrochemical analysis of exhaled breath condensate (EBC) from healthy human and animal subjects is reported on. EBC is a biospecimen potentially rich in biomarkers of respiratory disease. The EBC pH was analyzed potentiometrically using a disposable polyaniline (PANI)-modified inkjet-printed (IJP) carbon electrode. Comparison measurements were performed using a commercial screen-printed carbon (SPC) electrode. The PANI-modified electrodes exhibited reproducible and near-Nernstian responses for pH values between 2 and 9 with slopes from −50 to −60 mV/dec. The PANI-modified IJP carbon electrode exhibited a faster response time and superior reproducibility to the modified SPC electrode. In proof-of-concept studies, the healthy human EBC pH was found to be 6.57 ± 0.09 and the healthy bovine EBC pH was 5.9 ± 0.2. All pH determined using the PANI-modified electrodes were in good agreement with the pH determined using a micro glass pH electrode. An RTube device was used to collect EBC from humans while a modified device was used to collect EBC from calves in the field. EBC volumes of 0.5–2 mL for 5–6 min of tidal breathing were collected from healthy animals. The pH of EBC from healthy calves (17 animals) depends on their age from 1 to 9 weeks with values ranging from 5.3 to 7.2. A distinct alkaline shift was observed for many animals around 20 days of age. The bovine EBC pH also depends on the ambient temperature and humidity at the time of collection. The results indicate that the PANI-modified IJP carbon electrodes outperform commercial SPC and provide reproducible and accurate measurement of pH across various biospecimen types.

## Introduction

Pathogen infections in domestic animals pose a threat to food production and supply, animal harm, and have repercussions for the environment and biodiversity.^1,2^ Cell culture, fluorescence antibody tests (blood analysis), enzyme-linked immunosorbent assays, and other molecular tests are commonly used for detecting viruses and bacteria associated with animal disease.^1^ The main drawbacks of these conventional techniques are that they generally are expensive, time-consuming, and require specialized equipment/instrumentation and skilled staff to conduct the tests.^1,2^ The agriculture industry would benefit by integrating cutting-edge diagnostic and disease detection and monitoring systems that are less costly, easier to use and field deployable.^1^ Electrochemical sensors and immunosensors are ideal for point-of-care and point-of-field diagnostic technologies. Electrochemical devices offer rapid response time, ease of miniaturization, and good sensitivity and selectivity.^3−5^ Basic research is needed to develop electrochemical sensors, biosensors, and immunosensors for use in animal health care and to translate these devices from assays in the laboratory to practical application clinically.

Bovine respiratory disease (BRD) is a general term for a severe respiratory illness affecting young calves, typically after transport to a feedlot or the dairy. It is the costliest disease affecting dairy and feedlot beef cattle in North America.^6^ It is estimated that producers lose over $1 billion annually on BRD prevention, treatment, and production loss in U.S. cattle populations.^7,8^ BRD is complex and multifactorial with a variety of physical and physiological stressors combining to predispose cattle ultimately to pneumonia. There are various bacterial and viral pathogens that cause or are associated with the disease.^9,10^*Mannheimia hemolytica*, *Pasteurella multocida*, *Histophilus somni*, and *Mycoplasma bovis* are the bacterial agents that have been most consistently implicated in BRD.^11−13^ Three major viruses associated with BRD are bovine respiratory syncytial virus, bovine herpesvirus type 1 (BHV-1), and bovine parainfluenza virus type 3 (BPIV3).^9,11−14^ Although BRD can occur in any age and class of cattle, it is more common in young animals under stressful conditions.

**Figure 1:**
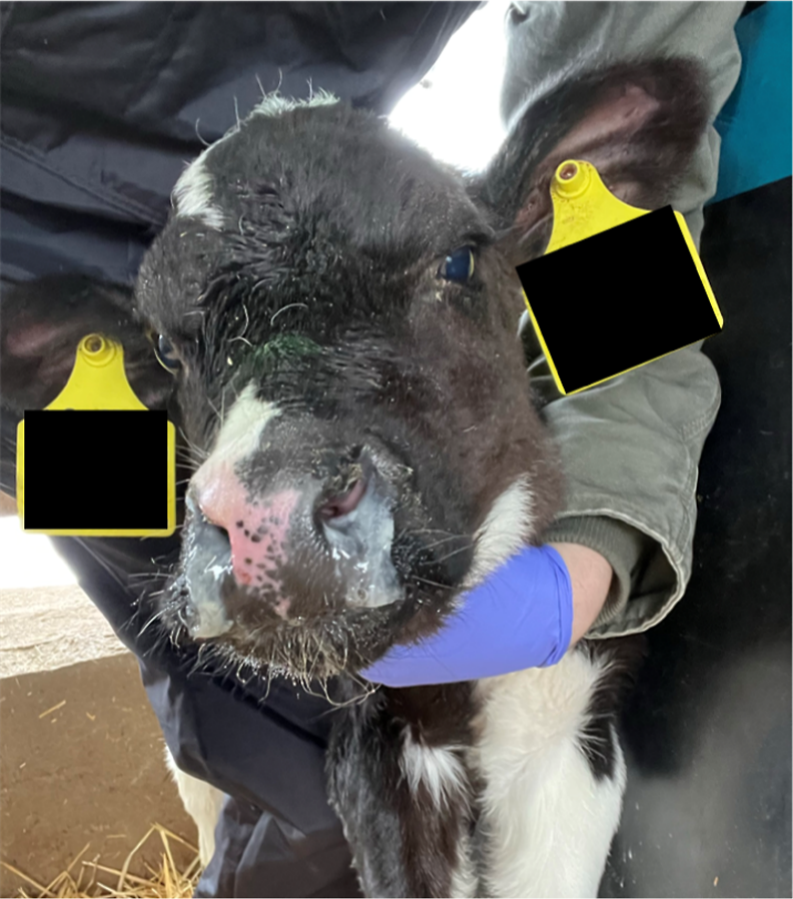
A calf, with excessive nasal discharge, is suspected of being ill with BRD.

BRD involves immunosuppression, respiratory infection with one or more pathogens, and culminates with bronchopneumonia caused by either exogenous bacteria or commensal bacteria that originate in the nasal pharynx.^9,15,16^ Clinical diagnosis of BRD in the feedlot is generally performed qualitatively using observational changes made in depression, appetite, respiration, and rectal temperature (DART).^2^ The first signs of illness usually involve reduction in appetite and lethargy.^17,18^ This may rapidly progress to a “drawn” appearance and an animal might become separated from the herd. Depression, drooped head and ears, nasal and ocular discharge, coughing/wheezing, and labored breathing are also symptoms of BRD. A suspected ill calf displaying some of these symptoms is shown in Figure 1. Once these signs are observed, the first line of treatment is isolation of the animal from the rest of the herd. There are laboratory tests available to detect pathogens involved in BRD. Samples from the respiratory tract can be cultured to identify bacterial pathogens and to determine sensitivity to antibiotics. Polymerase chain reaction testing methods can be used to quickly detect viral or bacterial BRD pathogens. Serological tests are also available to assess antibody levels toward various BRD pathogens as an aid in diagnosis. These tests are effective but are time-consuming and expensive. They also require invasively collected blood samples. Battling respiratory disease and limiting transmission through a herd could be better accomplished with less costly and more rapid analysis of noninvasively collected respiratory tract biospecimens. Furthermore, there is a need in the field to determine when an animal is no longer contagious and can be returned to the herd. An animal might still be contagious without exhibiting visible symptoms.

Biomarkers are substances that indicate a normal or abnormal biological state and can be used to detect or monitor a disease or health condition based on a change in their levels. They can be measured in blood, saliva, tears, sweat, urine, exhaled breath (EB), and exhaled breath condensate (EBC).^19^ EB can be collected noninvasively. EBC is the liquid phase of EB that is sampled by cooling. EBC contains proteins, small molecules (volatile and nonvolatile), and ions that originate from airway lining fluid (ALF).^20,21^ In principle, EBC composition reflects the biochemical profile of the ALF. The biochemical composition of ALF in healthy animals is expected to be distinctly different from that in calves infected with BRD. Furthermore, the biochemical composition of EBC should be distinctly different from that of EB, with the latter limited to small volatile molecules. EBC has been much less investigated as a reservoir of potential biomarkers in BRD than has EB.^2^ Therefore, research is warranted to investigate the usefulness of EBC as a biospecimen for diagnosing BRD and monitoring an animal’s progress during treatment.

There is no “gold standard” biomarker or suite of biomarkers identified for tracking the pathogenesis of BRD.^22^ In the disease state, airway inflammation and oxidative stress will develop in response to the pathogen. Therefore, it is expected that oxidative and nitrosative stress biomarkers will be at higher concentrations in EBC from ill animals and should trend toward the normal concentration range for healthy animals with successful treatment. During lung inflammation, oxidative stress mediators can be released into the ALF and may indicate the severity of the lung condition. Concentrations of oxidative and nitrosative molecules, such as hydrogen peroxide, nitric oxide, and peroxynitrite, should be elevated in the respiratory disease state and modulated by therapeutic treatment.^23−25^ The pH of EBC also changes in respiratory disease.^26−28^ Published work has collected EBC from horses and demonstrated that the pH and H_2_O_2_ levels in animals with lower airway inflammation are distinct from healthy controls.^29−31^ These biomarkers positively correlated with immune cells in bronchioalveolar lavage fluid. Similar EBC collection and testing approaches could be used to monitor BRD in bovine populations. Using EBC pH as a predictive marker of BRD has not been thoroughly validated.^2,32^ Some research has shown bovine affected with BRD tend to have higher white cell counts in blood (leukocytosis),^33^ increased serum content of haptoglobin,^34^ decreased EBC pH, and increased EBC hydrogen peroxide concentration, as compared to healthy controls.^33−35^

Since EBC pH has been identified as a potential biomarker of BRD, we fabricated polyaniline (PANI)-based potentiometric pH sensors for measuring the pH of bovine EBC. Conducting polymers, like PANI, are electrically insulating in the reduced form and conductive in the oxidized state.^31^ This process can be expressed as
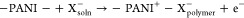
in which X_soln_^–^ is the charge compensating anion that enters the polymer upon oxidation. PANI exists in three oxidation states: leucoemeraldine, which is the fully reduced state; emeraldine base, which is the half-oxidized state; and pernigraniline, which is the fully oxidized state.^36^ The transition between the emeraldine salt and base due to the protonation/deprotonation of imine groups at different solution pH changes the polymer charge state and, consequently, its equilibrium potential. Relating this measured equilibrium potential to the solution pH enables direct and sensitive pH sensing.^37,38^ PANI is widely used in sensors due to its high conductivity, excellent redox recyclability, protonic dopability, chemical stability, low cost, and facile synthesis.^39^ Numerous published literature reports on the use of PANI-based sensors for measuring pH.^37,40−49^ PANI can be synthesized and doped by chemical or electrochemical methods.^50,51^ Electropolymerization is the most used method for the deposition of PANI films on electrodes due to its easy, fast, reproducible, cost-effective, and environmentally friendly properties.^50,51^ This synthesis is performed by anodic oxidation of the monomer on inert electrodes, such as gold, carbon, and platinum.^38^

Herein, we report on the field collection, processing, and pH analysis of bovine EBC. The objectives of this work were (i) to assemble and test a noninvasive sampling device for EBC collection from calves in the field, (ii) to validate EBC biospecimen transport, storage and processing conditions, and (iii) to compare the performance of PANI-modified electrochemical pH sensors. The EBC biospecimens were collected from cohorts of healthy and ill calves suspected of having BRD. PANI-modified inkjet-printed (IJP) nanocarbon electrodes were prepared as pH sensors and used to measure the EBC pH for both calf groups. The IJP electrodes were made with either carbon nanotube (CNT-IJP) or nanographene (Gr-IJP) inks. A polyacrylamide (PA) hydrogel was printed over the nanocarbon to form PA/CNT-IJP or PA/Gr-IJP electrodes, respectively. The innovative aspects of the work are (i) the validation of an EBC collection device for use with calves in the field, (ii) demonstration that the PANI-modified IJP sensors provide the best performance compared to the SPC counterparts and yield reproducible and accurate values of the pH across multiple types of biospecimens including bovine EBC, and (iii) showing that several physiological and environmental factors influence the EBC pH from healthy control animals.

## Methods and Materials

### Chemicals

All chemicals were high purity grade, or better, and were used as received (Sigma-Aldrich). A Britton–Robinson (BR) buffer stock solution was prepared by mixing boric acid (H_3_BO_3_, ≥99.5%), acetic acid (CH_3_COOH, 99.0%), and phosphoric acid (H_3_PO_4_, 85.0%), all at 0.5 M concentrations. The working buffer solutions were prepared by diluting the stock solution to 0.01 M and then adjusting the pH in the range of 2–9 with additions of a sodium hydroxide solution (NaOH, ≥97%). The electrodes were electrochemically modified with 0.1 M aniline (≥99.5%) in 0.1 M sulfuric acid (H_2_SO_4_, ≥95.0%) to form the PANI sensing layer. The salts used for the interference studies were sodium chloride (NaCl, ≥99.0%), potassium chloride (KCl, ≥99.0%), and calcium chloride (CaCl_2_·2H_2_O, ≥99.0%). All solutions were prepared with ultrapure water pressure filtered to 18.2 MΩ cm.

### Screen-Printed and Inkjet Printed Carbon Electrodes

The screen-printed carbon (SPC) electrodes were obtained from a commercial supplier and consisted of a carbon powder working electrode (4.0 mm in diam., geometric area of 0.126 cm^2^), a carbon powder counter electrode, and silver/silver chloride reference electrode (Metrohm, #DRP-C11L). The fully IJP carbon electrodes were prepared as individual working electrodes by a multistep printing process using a Fujifilm Dimatix DMP-2831 inkjet printer and DMC-11610 cartridges (10 pL nominal droplet volume). Details of the CNT ink-based electrode preparation have been published elsewhere.^52−54^ For the nanographene (Gr)-based electrode preparation, a graphene ink with ethyl cellulose in cyclohexanone and terpineol (Sigma-Aldrich) was used. The SunTronic U6415 UV curing jettable insulator ink (Sigma-Aldrich) was applied to print an insulation layer for defining accurately the geometric area of the working electrode (0.01 cm^2^). Both were applied after printing an underlying Ag contact pad and contact strip Ag dispersion in triethylene glycol monomethyl ether, (Sigma-Aldrich). A Kapton (polyimide) sheet (Müller Ahlhorn) was used as the flexible substrate on which the electrodes were printed. All printing parameters, such as the printing resolution (drops per inch, dpi), jetting voltage, and jetting speed were optimized in prior work. After printing the nanographene ink, the electrodes were heated to 400 °C to evaporate the ink solvent and stabilizer. The UV curable ink was printed and simultaneously cured using UV light from a curing wand in fixed position behind the print head. The light was generated from an Omnicure S2000 mercury lamp (Excelitas Technologies) and piped to the printer head using a liquid light guide. A PA hydrogel was IJP onto both the CNT and the Gr electrodes according to a procedure described in a prior publication to prepare PA/CNT-IJP and PA/Gr-IJP electrodes, respectively.^53^ In brief, a self-made ink containing the monomer acrylamide (Acros), cross-linker *N*,*N*′-methylenebis(acrylamide) (Acros), catalyst *N*,*N*,*N*′,*N*′-tetramethyl ethylenediamine (Sigma-Aldrich) and surfactant Triton X-100 (Sigma-Aldrich) was printed and quasi-simultaneously photopolymerized using the UV lamp of the printer setup. No PA hydrogel coating was applied to the commercial SPC electrodes.

### Instrumentation and Material Characterization

PANI synthesis by the polymerization of aniline was performed chronoamperometrically using a Gamry Instruments potentiostat/galvanostat (Reference 600, Warminster, PA). Cyclic voltammetry (CV) and open-circuit potential (OCP) measurements of standard solutions and biospecimens were made using a CH Instruments electrochemical workstation (model 650A, Austin, TX). Scanning electron micrographs (SEM) were obtained with a JEOL 7500F microscope (Tokyo, Japan). For SEM, the PA/CNT-IJP and PA/Gr-IJP electrodes were fully immersed in liquid nitrogen for 5 min and freeze-dried with an EMS750X freeze drier (Electron Microscopy Sciences, Hatfield, PA) under vacuum during a temperature program ramp from −70 °C to room temperature over a 24 h period. The electrodes were then coated with ca. 3 nm of osmium using a NEOC-AT CVD coater (Meiwafosis Co., Osaka, Japan). The SPC electrodes were coated with iridium for 60 s under an Ar gas environment using a Q150T turbo-pumped sputter coater (Quorum Technologies, Sacramento, CA). These thin metal coatings minimized surface charging on the carbon electrodes and enabled the acquisition of better-quality micrographs. All microscopes were maintained in the Michigan State University (MSU) Center for Advanced Microscopy.

Voltammetric and amperometric measurements were performed by immersion in a single compartment glass electrochemical cell containing the appropriate solution. IJP electrochemical measurements were made in a three-electrode conformation with a PA/nanocarbon-IJP working electrode, a platinum wire counter electrode, and a mini-Ag/AgCl (3 M KCl) reference electrode (eDAQ, #ET073, Colorado Springs, CO). For SPC electrodes, these electrochemical measurements were made by immersing the screen-printed, three-electrode assembly into the same single-compartment glass electrochemical cell.

### Electrodeposition of PANI

**Figure 2:**
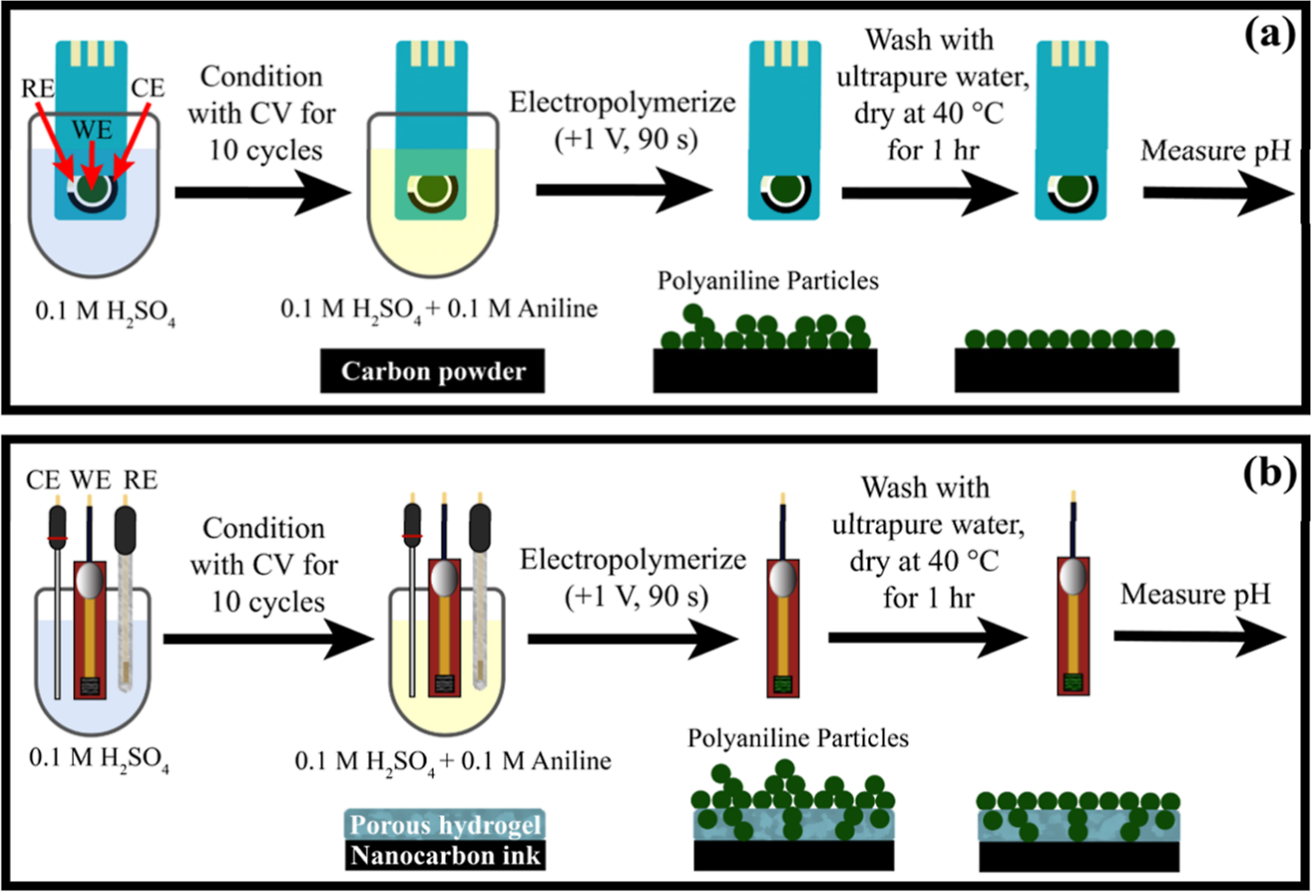
Processing steps for PANI electrodeposition on (a) screen-printed (SPC) and (b) IJP electrodes from a solution of 0.1 M aniline in 0.1 M H_2_SO_4_.

PANI was electrodeposited on the carbon electrodes by controlled potential electrolysis according to the method described by Mazzara and co-workers,^41^ with some slight modifications. The steps performed for IJP and SPC electrode modification are indicated in Figure 2. Prior to electrodepositing the conducting polymer, the IJP or SCP working electrode was placed in 0.1 M H_2_SO_4_ and cycled 10 times for conditioning between −0.20 and 0.60 V for the PA/CNT-IJP electrode, −0.10 and 0.70 V for the PA/Gr-IJP electrode and −0.40 to 0.40 V for the SPC electrode. For the IJP electrodes, all potentials are referenced against an Ag/AgCl electrode (3 M KCl). For the SPC electrodes, the potentials are reported against a quasi-Ag/AgCl reference electrode (see below). The potential windows for conditioning were chosen based on PANI redox potential ranges on each electrode (see Figure 4b). The electrodes were then modified with PANI by immersing the electrodes in 0.1 M aniline dissolved in 0.1 M H_2_SO_4_ and applying +1.0 V vs Ag/AgCl (3 M KCl) for 90 s to prepare PANI/PA/CNT-IJP, PANI/PA/Gr-IJP, and PANI/SPC electrodes. After the deposition, the PANI-modified working electrode was gently rinsed with ultrapure water and dried at 40 °C for 1 h. Thereafter, CV (5 scans) was performed in 0.1 M H_2_SO_4_ over the same potential ranges listed above to condition the modified electrode and confirm PANI deposition.

### PANI Electrode Response and Biospecimen pH Measurements

Each PANI-modified electrode was soaked in ultrapure water for 20 min to hydrate the polymer prior to any pH measurement. PANI/PA/nanocarbon-IJP electrodes were immersed with a mini-Ag/AgCl (3 M KCl) reference electrode in a centrifuge tube containing the 0.01 M BR buffers with pH ranging from 2 to 9 and the equilibrium rest potential recorded. Response curves were generated by plotting the equilibrium potential (*E*) vs solution pH. This was repeated for the PANI/SPC electrodes using the Ag/AgCl reference on the platform. Rather than immersing the PANI/SPC, 60 μL of BR buffer was dropped on the three-electrode configuration printed on the substrate. BR buffer pH was measured using a standard combination micro glass pH electrode (Thermo Fisher Scientific, Orion 8220BNWP PerpHecT ROSS, Waltham, MA) coupled to an Orion Star A111 pH meter (Thermo Fisher Scientific, Waltham, MA). The micro glass pH electrode was calibrated with standard buffers at pH 4.01, 7.00, and 10.01 before use. Biospecimen pH measurements were made with the PANI/PA/Gr-IJP and PANI/SPC electrodes in the same way as above but with the buffer being replaced with biospecimen analyte solution. The resulting working electrode potential was compared to the *E* vs pH response curve generated using BR buffers to determine the biospecimen pH. All electrochemical measurements were performed at room temperature (ca. 23 °C). All biospecimen pH values were validated with the same micro glass pH electrode listed above.

### Biospecimen Collection and Processing

EBC was collected from human volunteers using an RTube Breath Condensate Collection Device (Respiratory Research, Inc., Austin, TX) after at least 1 h of fasting from food and drink. In this FDA-approved device, exhaled air is directed through a one-way valve into a cooled collection tube where volatiles, aerosol particles and moisture condense along the inside tube wall. An aluminum sleeve was stored on dry ice (−80 °C) for ≥30 min. During sampling, the cold sleeve was placed over the device’s collection tube. EBC was collected on multiple days from the same two healthy human volunteers (male and female) during tidal breathing for 3–5 min. The EBC collection rate was approximately 200 μL min^–1^. After collection, the tube was removed from the mouthpiece and one-way valve, capped, and prepared for immediate analysis by the following steps. The biospecimen condensate was pooled on an aluminum rod that slid along the inside wall of the collection tube from the bottom up. The liquid EBC was then collected with a micropipette and transferred into a 1.5 mL polypropylene centrifuge tube (Eppendorf). The EBC biospecimen was then centrifuged at 7000 rpm for 15 min. The capped biospecimen was then allowed to stand for 15 min to allow any generated aerosol particles to settle. The biospecimen centrifugate was then removed with a disposable syringe through a 0.22 μm PVDF membrane filter and transferred into a clean centrifuge tube. EBC pH was recorded directly in this filtered biospecimen.

Saliva biospecimens were collected from a single human volunteer (male) after at least 1 h of fasting from food and drink. Approximately 0.5 mL of saliva was collected directly into a 1.5 mL centrifuge tube. The biospecimen was centrifuged and filtered as done for the human EBC. pH measurements were made directly on the saliva in the centrifuge vial using both the PANI/PA/Gr-IJP electrode and micro glass pH electrode.

**Figure 3:**
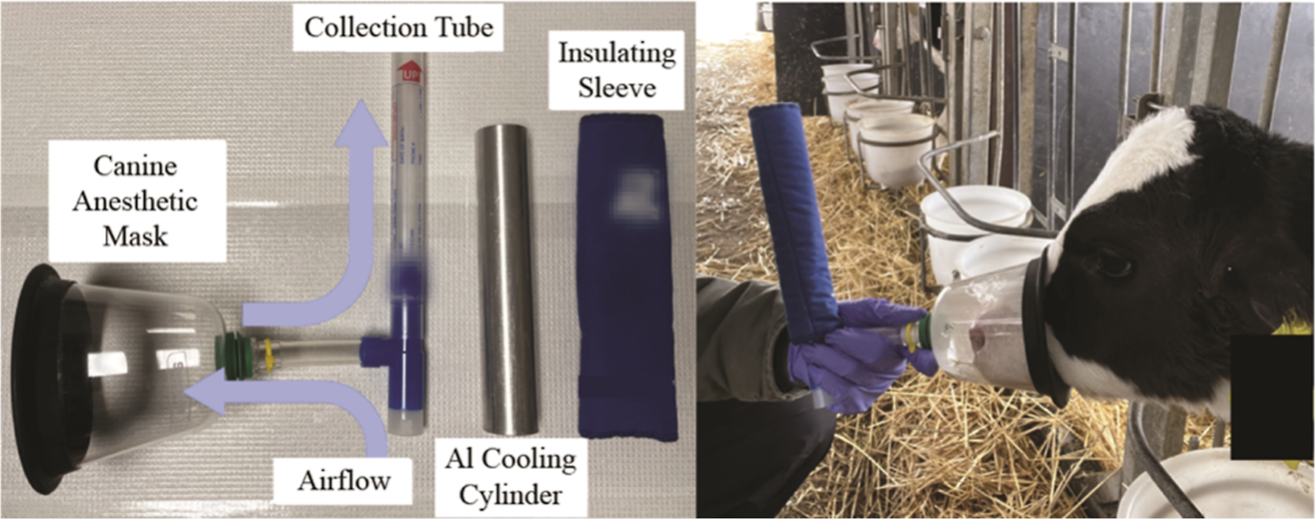
Apparatus used to collect the EBC biospecimens from calves in the field.

Bovine EBC was collected from calves in the field using a homemade device that consisted of a SurgiVet anesthesia mask coupled with to an RTube collection device. The apparatus and its application in the field are shown in Figure 3. In the process, animals exhale through the mask and into the collection device, as indicated by the upward arrow. As the animal breathes normally through a mouthpiece and one-way value, the device gathers breath condensate in a cooled collection tube. EBC was collected for 5–6 min producing 0.5–2 mL of condensate, depending on the animal, its age, and the ambient collection conditions. The collection tube was then removed, capped, labeled, and placed on dry ice for transport back to the laboratory. The anesthesia mask was removed from the tee-valve, sanitized with pure ethanol, and dried between collections from different animals. A new specimen collection tube was used for each sampling. All healthy calves were housed in outdoor hutches under ambient conditions at the MSU Dairy Cattle Teaching & Research Center (East Lansing, MI) and displayed no signs of illness. The calves ranged in age from 6 to 63 days old (mean ± std. dev. = 33 ± 17 days, median = 34 days). Ill calves suspected of having BRD were housed in a partially enclosed and ventilated barn at a commercial dairy farm in the Lansing, MI area. The calves were screened for illness based on coughing, nasal discharge, and lethargy. Calf EBC was then collected from animals with labored breathing and/or elevated rectal temperature (≥40 °C). Ill calves ranged in age from 22 to 70 days old (mean ± std. dev. = 38 ± 12 days, median = 38.5 days). The calf EBC biospecimens were processed for analysis in the same way as the human EBC biospecimens described above. All calves sampled from were female.

The wound swab biospecimen was collected from a canine subject being treated at the Small Animal Veterinary Clinic at MSU. This was done by rolling a sterile cotton tip applicator in a zigzag pattern across the wound surface after clipping the wound edges and then cleaning with a surgical scrub of chlorhexidine and sterile saline to limit contamination. After collection, the cotton tip was placed in 0.5 mL of ultrapure water in a 1.5 mL centrifuge tube and immediately frozen at −80 °C. To record the wound medium pH, the biospecimen was thawed for 20 min, centrifuged at 7000 rpm for 15 min, and allowed to stand in the capped centrifuge tube for 1 h to allow any generated aerosol particles to settle. The centrifugate was then passed through a 0.22 μm PVDF membrane filter attached to a disposable syringe and transferred to clean 1.5 mL centrifuge tube for immediate pH measurement.

The calf EBC and wound swab biospecimen collection procedures were approved by the Institutional Animal Care and Use Committee (IACUC) at MSU according to procedures PROTO 202200146 and PROTO 202000213.

## Results

### Electropolymerization of Aniline to Deposit PANI on IJP Electrodes

**Figure 4:**
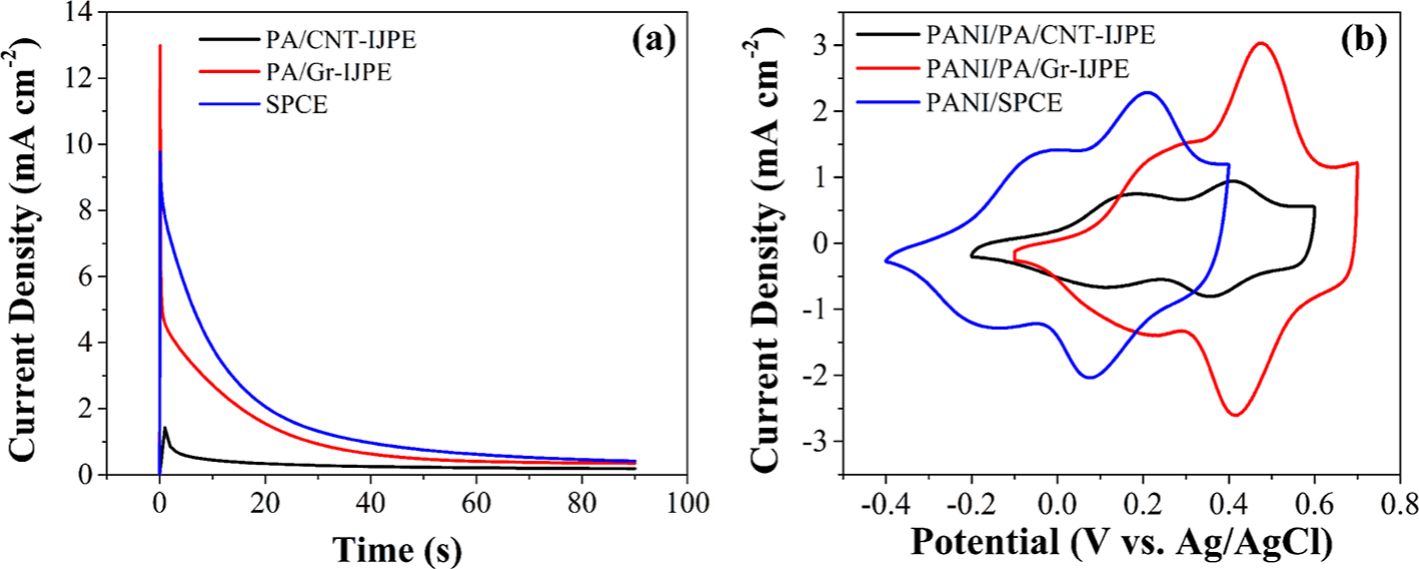
Representative (a) chronoamperometric *i*–*t* curves for the electrodeposition of PANI by constant potential electrolysis in 0.1 M aniline dissolved in 0.1 M H_2_SO_4_ and (b) cyclic voltammetric *i*–*E* curves for PANI-modified PA/CNT-IJP (black) and PA/Gr-IJP (red) electrodes in 0.1 M H_2_SO_4_. Comparison measurements for the SPC electrodes are also presented (blue). Reference = Ag/AgCl (3 M KCl) for the IJP electrodes and a quasi-Ag/AgCl for the SPC electrode. Application of +1.0 V for 90 s. Scan rate = 50 mV s^–1^.

Representative chronoamperometric *i*–*t* curves recorded during the potentiostatic deposition are presented in Figure 4a. The current density increases initially at both the PA/CNT-IJP and PA/Gr-IJP electrodes upon application of the potential step and then decays at short times afterward due to charging of the electric double layer. The current eventually reaches a steady state consistent with a constant rate of aniline radical cation formation, radical coupling, and PANI growth. This steady-state current density reached after 40 s is ca. 0.25 μA cm^–2^ for the PA/CNT-IJP electrode and after 50 s is ca. 0.5 μA cm^–2^ for the PA/Gr-IJP electrode (Figure 4a). The oxidation current density is slightly higher for the SPC electrodes reaching a steady state after 60 s of ca. 0.6 μA cm^–2^. The major difference is the larger current density for the SPC electrode. Recall, the larger geometric area of the SPC electrodes (0.126 cm^2^) and the fact that the smaller geometric area IJP electrodes (0.01 cm^2^) were also coated with a nanoporous PA hydrogel. According to Faraday’s law, the charge passed is controlled by the number of aniline radical cations formed and therefore is an indirect measure of the amount of polymer formed.^26^ Integrating the area under the *i*–*t* curves for the three electrodes yielded charge values (mean ± std. dev. for *N* = 3 electrodes of each type) of 0.25 ± 0.01 mC for the PA/CNT-IJP electrode, 1.05 ± 0.07 mC for the PA/Gr-IJP electrode, and 16.2 ± 1.8 mC for the SPC electrode. Dividing the charge by the geometric area of each electrode yields charge densities of 25 ± 1 mC cm^–2^ for the PA/CNT-IJP electrode, 105 ± 7 mC cm^–2^ for the PA/Gr-IJP electrode (4× larger), and 130 ± 14 mC cm^–2^ for the SPC electrode (5× larger).

After thorough rinsing with ultrapure water and oven-drying, cyclic voltammetric *i*–*E* curves were recorded for the PANI-modified IJP and SPC electrodes in 0.1 M H_2_SO_4_. Representative curves are presented in Figure 4b. The electrochemical behavior of PANI is similar on both IJP electrodes (black, red) and on the comparison SPC electrode (blue). PANI can exist as a salt or base in three isolable oxidation states: (1) leucoemeraldine; the fully reduced state, (2) emeraldine; the half-oxidized state, and (3) pernigraniline; the fully oxidized state. The partially and fully oxidized forms of the polymer represent the conducting states with charge carriers electrogenerated in the polymer.^55^ For the PANI/PA/CNT-IJP electrode, the leucomeraldine/emeraldine redox couple is observed at ca. 0.15 V, and the emeraldine/pernigraniline redox couple is at ca. 0.38 V. Similarly, for the PANI/PA/Gr-IJP electrode, the peaks are well-defined with the leucomeraldine/emeraldine redox couple at ca. 0.25 V, and the emeraldine/pernigraniline redox couple at ca. 0.45 V.^56^ Well-defined redox transitions are seen for PANI on the SPC electrode as well, although the potentials of both redox pairs are shifted slightly negative of the values for PANI on the IJP electrodes. The leucoemeraldine/emeraldine redox couple is centered at ca. −0.10 V, while the emeraldine/pernigraniline redox couple is observed at ca. 0.14 V. This difference in potentials results because of the two reference electrodes used. For the IJP electrodes, a separate thermodynamic Ag/AgCl (3 M KCl) reference electrode was employed while the Ag/AgCl reference electrode on the SPC electrode was a quasi-reference electrode developing a unique reference potential in the H_2_SO_4_ electrolyte solution devoid of chloride ions. Similar PANI voltammetric behavior was observed for multiple electrodes of each type and the current response was stable over the 5 cycles tested.

### Characterization of the PANI-Modified Electrodes

**Figure 5:**
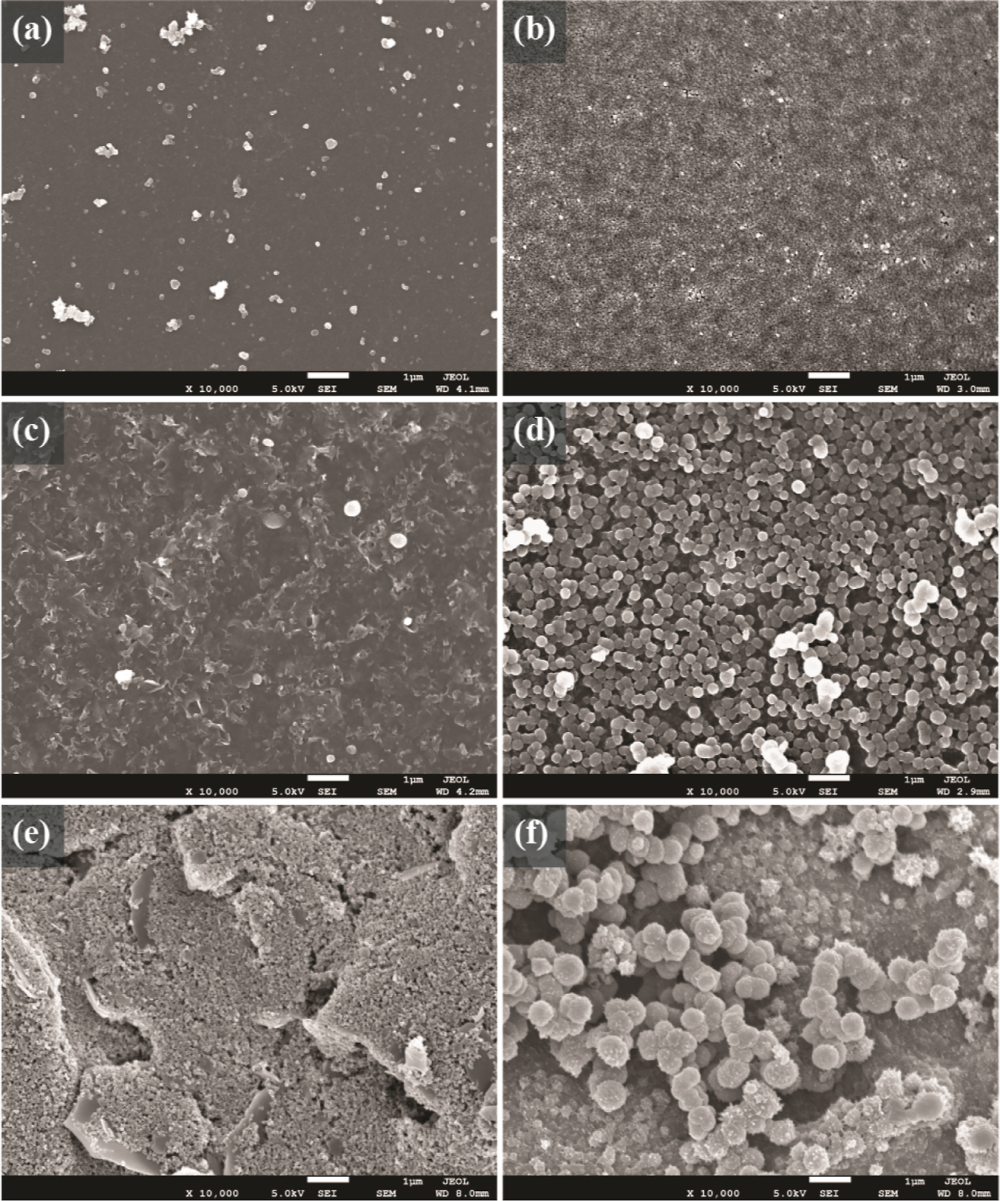
Scanning electron micrographs of a representative PA/CNT-IJP electrode (a) before and (b) after modification with PANI, and a representative PA/Gr-IJP electrode (c) before and (d) after modification with PANI. For comparison, scanning electron micrographs are presented for a representative SPC electrode (e) before and (f) after modification with PANI. All micrographs were collected using secondary electron mode and are presented at 10,000× magnification.

The PA/CNT-IJP and PA/Gr-IJP electrode surfaces were characterized using scanning electron microscopy before and after polymer formation. Comparison micrographs are also recorded for an SPC electrode before and after polymer formation. Representative micrographs of each electrode are presented in Figure 5. The micrographs for the PA/CNT-IJP electrode before and after PANI modification (Figure 5a,b) reveal a thin, uniform coverage of polymer particles over the hydrogel surface. Prior work revealed the nominal pore diameter of the hydrogel to be ca. 13 nm.^53^ The PANI forms on the electrode as individual islands within and outside of each pore. In some regions, polymer growths from neighboring pores have enlarged to the point of coalescence. A different polymer morphology with greater coverage is seen on the PA/Gr-IJP electrode (Figure 5c,d). We surmise the exposed nanographene particles are more active for the oxidation of aniline and this leads to the larger particle size and greater coverage. This is consistent with the greater charge observed in the chronoamperometric and cyclic voltammetric data for the PANI/PA/Gr-IJP electrode presented in Figure 4. PANI tends to form larger spherical particles that agglomerate to form a less uniform film over the hydrogel surface than the PA/CNT-IJP. The agglomerates that are generally spherical in shape form on both IJP electrodes with dimensions in the range of 100–500 nm.

Figure 5e,f present electron micrographs for the SPC and PANI/SPC electrodes. For the SPC electrode, the electrode morphology consists of a thick three-dimensional layer of nanocarbon powder particles mixed with ink or diluent. There are also void regions such as those in the lower left of Figure 5e. These are regions where analyte solution could penetrate or PANI formation could occur within the interior of the electrode. These types of voids or defects are not seen on the IJP electrodes, which are more two-dimensional in their morphology. PANI forms across the entire SPC electrode with a nodular morphology consisting of spherical particle aggregates, as seen in Figure 5f. The polymer particles are larger on the SPC than on the IJP electrodes in the range of 400–800 nm reflective of more extensive polymer growth on the former. The agglomerates tend to form on carbon powder ridges where more edge plane carbon sites likely are available for polymer nucleation. The polymer then grows from these nuclei to fill the three-dimensional carbon structure.

### Calibration Curves for PANI-Modified Electrodes

**Figure 6:**
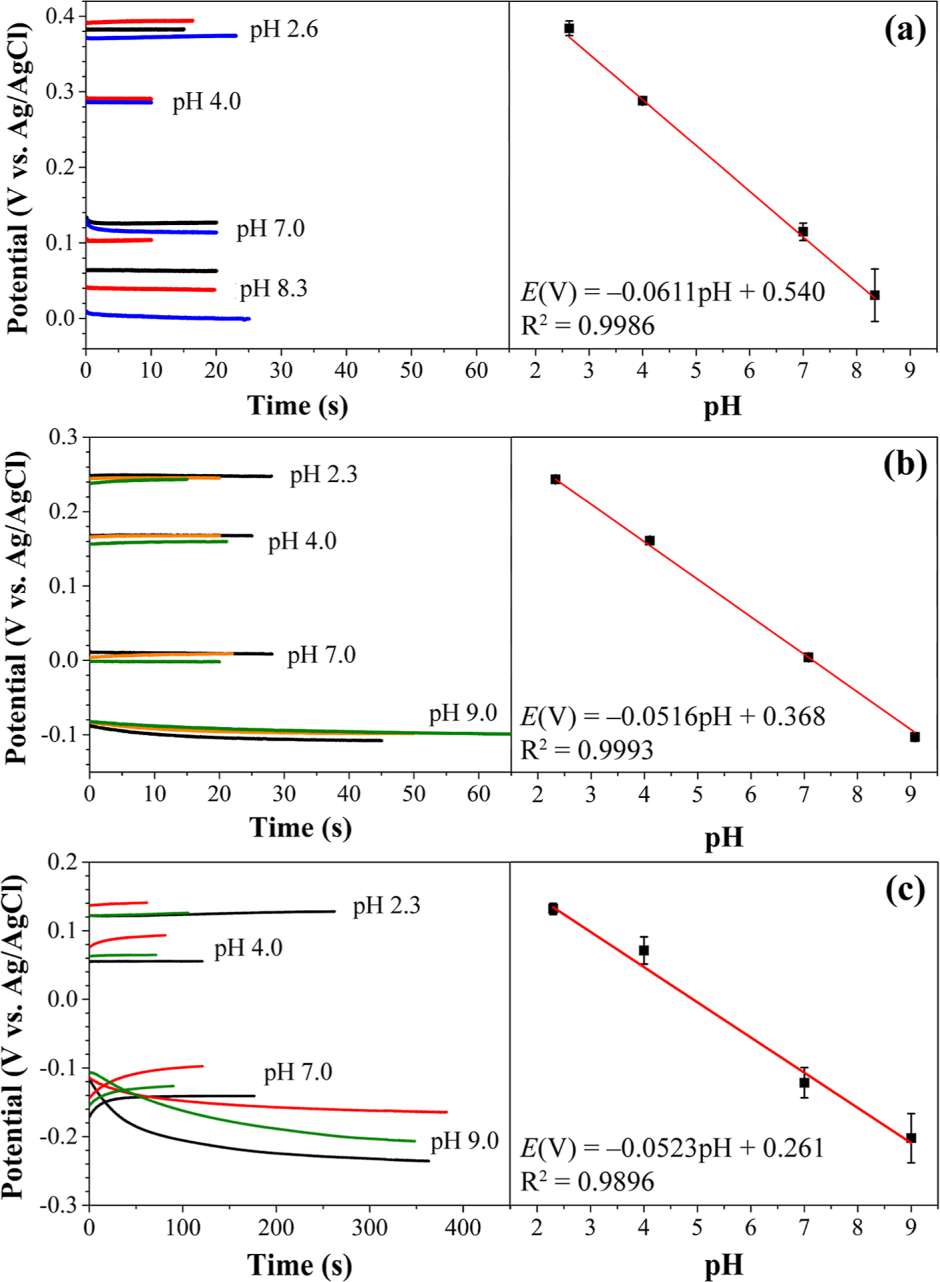
Potential vs time measurements in standard 0.01 M BR buffer solutions ranging between pH 2.3–9.0 using multiple (a) PANI/PA/CNT-IJP and (b) PANI/PA/Gr-IJP electrodes. Comparison measurements with the PANI/SPC electrodes are also presented (c). Corresponding potential vs pH response curves are displayed on the right. Data are presented in the response curves as mean ± std. dev. values for *N* = 3 sensors of each type in all four buffer solutions.

The performance of the PANI/PA/CNT-IJP and PANI/PA/Gr-IJP electrodes for pH measurements using standard buffer solutions was evaluated initially. Comparison measurements using the PANI/SPC electrodes were made. The measurements used standard BR buffer solutions in the pH range of 2–9. Figure 6 shows potential–time curves for multiple sensors in each buffer and compiled data in the form of potential vs pH response curves. Slope values below are reported as mean ± std. dev. for *N* = 3 representative sensors. Figure 6a reveals that for the PANI/PA/CNT-IJP electrodes, the potential stabilized rapidly within 10 s at all the pH values. The response curve slope was −61 ± 5 mV pH^–1^ (*R*^2^ = 0.9986), which is in accordance with the expected Nernstian value. Figure 6b reveals the PANI/PA/Gr-IJP electrodes achieved a stable potential after about 60 s for pH 9.0, and 20 s for pH 2.3–7.0. The sensors exhibited a near-Nernstian slope of −52 ± 0.7 mV pH^–1^ (*R*^2^ = 0.9993). All electrodes exhibited good reproducibility as evidenced by the small error bars, except in the pH 9.0 solution. At low pH, the conductivity of PANI is high due to the proton doping process, thus the redox peaks are better defined. As the pH increases, the PANI film becomes more deprotonated and its conductivity decreases due to the loss of internal charge states (i.e., protonated amine sites with counterbalancing anions). Therefore, the voltammetric redox peak is broader, which leads to a less reproducible potential.^37^ The PANI/PA/Gr-IJP electrodes exhibited the better response reproducibility of the two IJP electrodes. Comparison measurements with the PANI/SPC electrodes, presented in Figure 6c, revealed stable potentials after approximately 300 s for pH 9.0 and 7.0, and approximately 120 s for pH 4.0 and 2.3. The slope of the calibration curve for multiple sensors was −52 ± 4 mV pH^–1^ (*R*^2^ = 0.9896). The longer time to reach equilibrium for the PANI/SPC electrodes is attributed to the greater polymer coverage and a thicker PANI layer.^42^

The pH response mechanism of the PANI-modified sensors arises from the proton doping process within the polymer changing it from the base tothe salt form, according to eq 1.^37^
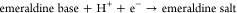
1

Since the reduction reaction is proton-dependent, the pH values of the analyzed buffers can be correlated with the equilibrium potential (*E*) measured according to the Nernst eq (eq 2).
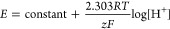
2*R* is the universal gas constant (8.314 J K^–1^ mol^–1^), *T* is the temperature (295 K), *z* is the charge of the proton, and *F* is the Faraday constant (96,485 C mol^–1^).^41^ Inserting numerical values in the equation, converting to log_10_, and considering that pH = −log[H^+^], eq 3 is obtained.
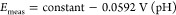
3

Therefore, it is expected that a plot of *E*_meas_ vs pH should be linear with a theoretical slope of −59 mV pH^–1^.^45,57^

### Evaluation of the PANI Sensor Reproducibility and Reusability

The reusability of the PANI-modified electrodes after short-term storage was evaluated. For this study, the same electrode (only one of each type) was used to measure the pH of 0.01 M BR buffer solutions at pH 2.3, 4.0, 7.0, and 9.0 on three consecutive days. The slopes of the *E*_meas_ vs pH plots recorded each day were compared. Between measurements, the sensors were rinsed with ultrapure water and stored in the laboratory atmosphere at room temperature (23 °C) in a covered Petri dish. For the PANI/PA/CNT-IJP electrode, the slope decreased by 7 mV pH^–1^ on day 2 and increased by 1 mV pH^–1^ on day 3, relative to day 1, giving an RSD of 7.3%. The response time was short and remained unchanged on all 3 days. For the PANI/PA/Gr-IJP electrode, the slope decreased by 2 mV pH^–1^ on day 2 and increased by 5 mV pH^–1^ on day 3, relative to day 1, giving an RSD of 5.1%. Also, the response time was short and unchanged on all 3 days. Overall, the results for the PANI/IJP sensors indicate the interday reproducibility is good and that short-term storage in air does not significantly alter the sensor response. The slight variation in daily sensitivity observed for all the sensors is attributed to very minor changes in the number of amine sites available for protonation.^44^ For the PANI/SPC electrode, the slope decreased by 5 mV pH^–1^ on day 2 and then increased by 2 mV pH^–1^ on day 3, relative to day 1, giving an RSD of 5.6%. Overall, the reproducibility for standard solutions and short-term stability of the PANI-modified sensors are good for all three carbon electrode types.

The reusability and reproducibility of the electrodes after long-term storage were evaluated. The *E*_meas_ vs pH response of the PANI/Gr-IJP electrodes was measured on days 0, 7, 21, and 29 using the same procedure as the short-term reproducibility study above. Results are presented as mean ± std. dev. (% RSD) for *N* = 3 electrodes. On day 0, the PANI/Gr-IJP response was −51.0 ± 0.3 mV pH^–1^ (0.6%). On days 7, 21, and 29, the response was 52.1 ± 1.0 (1.9%), −53.3 ± 0.8 (1.5%), and −54.0 ± 0.6 mV pH^–1^ (1.1%), respectively. These results show that the PANI/Gr-IJP electrodes exhibit a stable and reproducible response over about a month of storage and reuse.

### Hysteresis Effects

**Figure 7:**
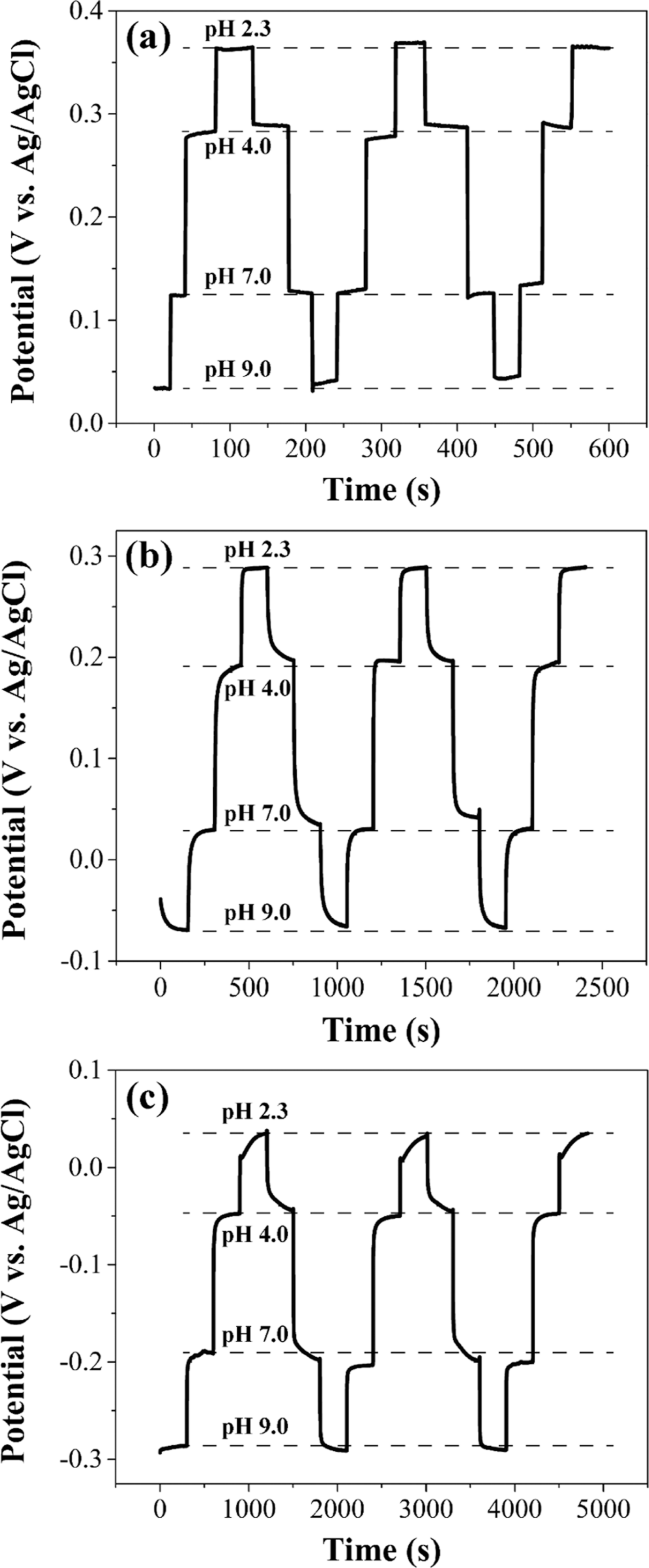
Repeatability tests in buffer solutions of different pH without any cleaning step between each measurement for (a) PANI/PA/CNT-IJP, (b) PANI/PA/Gr-IJP and (c) PANI/SPC electrodes. Plots of the equilibrium potential vs time during contact with the different buffer solutions are presented.

Hysteresis or “memory effect” relates to the PANI electrode response when placed in solutions of differing pH without any cleaning step between the measurements.^46^ To evaluate this, the OCP of a sensor was successively measured in buffers (ca. 20 mL volume) of differing pH by immersion without any rinsing or cleaning step between each measurement. The results are presented in Figure 7. The equilibrium potential was achieved for the PANI/PA/CNT-IJP electrode within 25 s (Figure 7a), for the PANI/PA/Gr-IJP electrode within 150 s (Figure 7b), and for the PANI/SPC electrode by 300 s in each solution (Figure 7c). The maximum slope differences between subsequent hysteresis groupings (i.e., measuring pH 9.0 to 2.3 and then 2.3 to 9.0) for all three electrode types were only 0.7, 0.6, and 0.7 mV pH^–1^, respectively. The data indicates that the same PANI-coated electrode can be used for multiple measurements with limited carry over effects.

### Influence of Interfering Ions

**Figure 8:**
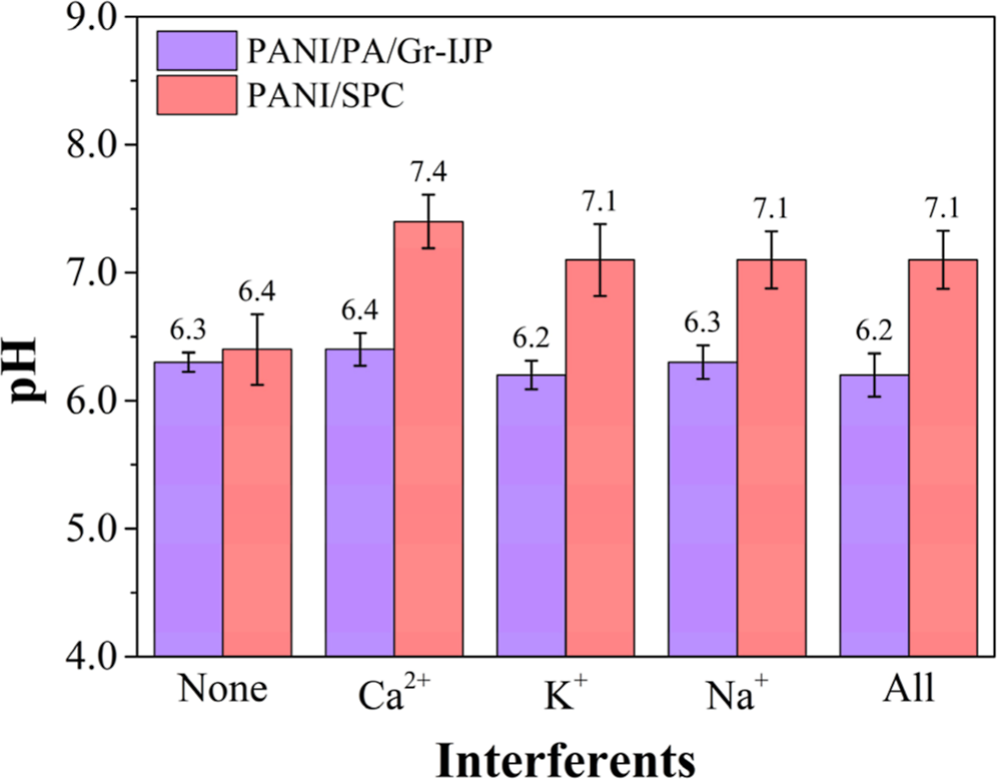
Influence of 38 μM of Na^+^, 9 μM of Ca^2+^ and 11 μM of K^+^ on the pH determination of a 0.01 M BR buffer at pH 6.40 using the PANI/SPC and PANI/PA/Gr-IJP electrodes. Data are presented as mean ± std. dev. for *N* = 3 electrodes of each type. The PANI/PA/Gr-IJP electrode pH results in the presence of cations were not statistically different to the control as assessed a two-tailed, paired student’s *t*-test with a difference assessed at *p*-value ≤0.05.

To assess the selectivity of the PANI/IJP and PANI/SPC electrodes for measuring the hydrogen ion concentration in solution, the influence of some possible interfering cations was investigated. For these measurements, a 0.01 M buffer solution at pH 6.45 was spiked with 38 μM of Na^+^, 9 μM of Ca^2+^, and 11 μM of K^+^, using NaCl, CaCl_2_, and KCl salts, respectively. The ion concentrations were selected based on a literature study in which the authors compared the concentrations of different ions detected in EBC biospecimens from healthy humans before and after exercising.^58^ The pH of the buffer was set at 6.45 to be close to the EBC pH value often measured in healthy humans.^58,59^ The measured pH obtained using the buffer without any of the interfering ions, with each ion present individually, with all the ions present are compared in Figure 8. The pH of the buffer without the interfering ions was measured before and after the sequence of interfering cations. For the PANI/SPC electrode, all ions affected the pH measurement increasing it about 0.5 to 1.0 pH unit, especially for Ca^2+^. The PANI/PA/Gr-IJP electrode pH readings, on the other hand, were not affected by the interfering cations. A reason for the shift in potential for the PANI/SPC electrode is that the potential of the on-platform Ag/AgCl layer (i.e., reference electrode) shifts with the different concentrations of Cl^–^ from the interfering salt solutions. Importantly, the data suggests that the PANI/IJP electrodes can be used to provide reliable pH measurements in solutions of differing ionic composition.

### Measurement Human EBC Biospecimen pH

EBC biospecimens were collected from healthy human volunteers on three different days according to the procedure described in the Experimental Section. A summary of the pH data is presented in Table 1. The samples were analyzed using the PANI/PA/Gr-IJP and PANI/SPC electrodes. The determined pH values were validated by comparison against the values obtained using a micro glass pH electrode. Three sensors of each carbon type were reused to record the EBC pH on the different days. The nominal values obtained with both PANI carbon electrode sensors are similar and are in good agreement with the values returned with the micro glass pH electrode. The exception is the pH value determined with the PANI/SPC electrode on day 2. Notably, the standard deviations associated with the PANI/SPC electrode data are larger than those for the PANI/IJP electrode.

**Table 1:** Comparison of Human EBC pH Data Obtained Using the PANI/SPC and PANI/PA/Gr-IJP Electrodes with Values Obtained Using a Micro Glass pH Electrode^c^

	day 1	day 2	day 3
PANI/PA/Gr-IJP^a^	6.57 ± 0.09	6.4 ± 0.1	6.08 ± 0.03
micro pH electrode^a^	6.3	6.2	6.0
PANI/SPC^b^	6.6 ± 0.6	5.9 ± 0.1	6.4 ± 0.1
micro pH electrode^b^	6.6	6.4	6.4

a Values are presented as mean ± std. dev. for *N* = 3 measurements of each EBC biospecimen. The EBC biospecimens were collected on different days from a healthy male volunteer, age 23.

b The EBC biospecimens were collected on different days from a healthy female volunteer, age 29.

c The same PANI/PA/Gr-IJP and PANI/SPC electrodes were used for each of the three EBC biospecimens. All PANI-electrode results were not statistically different from the pH values determined using the micro glass electrode, as assessed using a two-tailed, paired student’s *t*-test with a difference assessed at *p*-value ≤0.05.

### Measurement of Bovine EBC Biospecimen pH

Given the superior properties of response time, sensitivity, reproducibility, and lower ion interference effects, the PANI/PA/Gr-IJP electrodes were used to record the pH of three additional biospecimens: bovine EBC, human saliva, and canine wound swabs. Processing steps for each biospecimen type are described in the Experimental Section. The summarized results in Table 2 reveal that there is good agreement between the pH readings from the PANI/PA/Gr-IJP and the micro glass pH electrodes. These results suggest the PANI sensors can be used to accurately measure the pH of different types of biospecimens.

**Table 2:** Comparison of Different Biospecimen pH Data Obtained Using the PANI/PA/Gr-IJP Electrodes along with Values Obtained Using a Micro Glass pH Electrode^a^

	bovine EBC	saliva	wound swab
PANI/PA/Gr-IJP	5.9 ± 0.2	7.5 ± 0.2	7.1 ± 0.1
micro glass pH electrode	5.8	7.4	7.2

a Results are reported as mean ± std. dev. for *N* = 3 measurements of each biospecimen type. All PANI-electrode results are statistically similar to the respective micro glass pH electrode value as assessed a paired student’s *t*-test with a difference assessed at *p*-value ≤0.05. The human saliva biospecimen was collected from healthy male volunteer, age 23. The bovine EBC was collected from a single female calf, age 3 weeks.

### Physiological and Environmental Variables Affecting the pH of Bovine EBC

**Figure 9:**
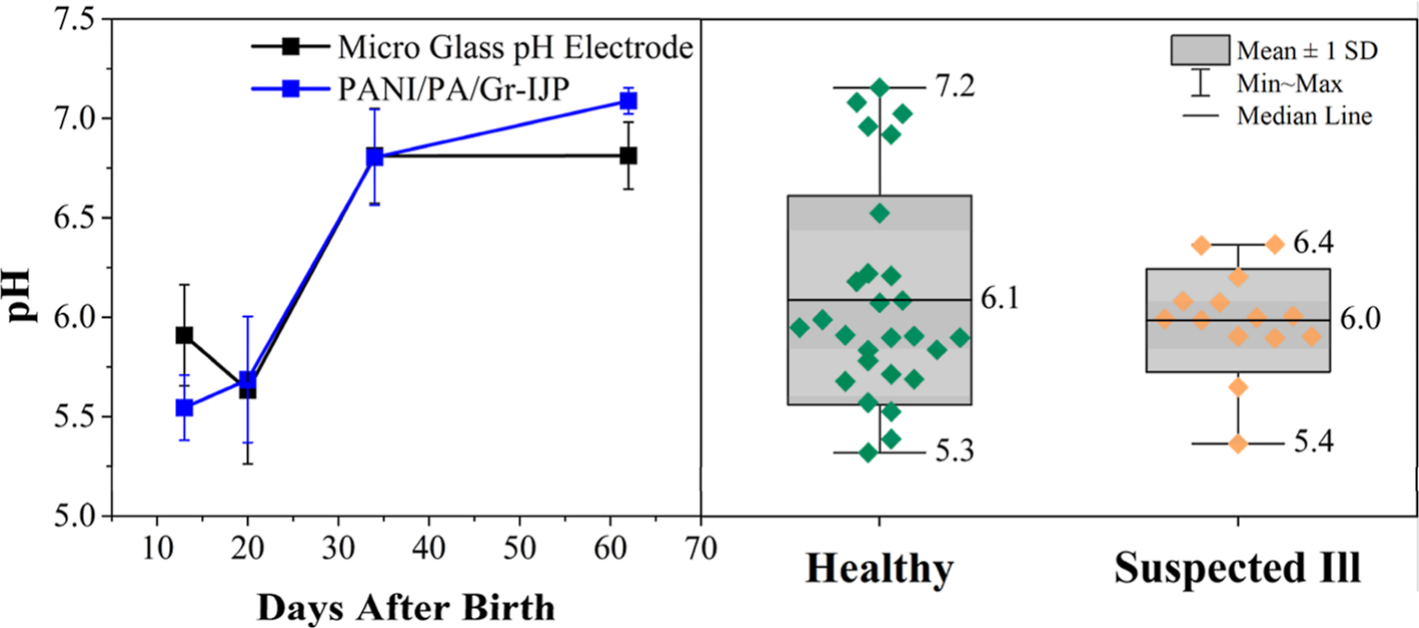
(a) EBC pH data for three healthy female calves as a function of age obtained using PANI/PA/Gr-IJP electrodes. pH data are also presented for the same biospecimens obtained using a standard micro glass pH electrode. Data are plotted as the average ± std. dev. for the three calves examined. (b) Plot showing the aggregate pH data for multiple healthy (6 animals, 27 measurements) and ill (14 animals) female calves obtained with the PANI/PA/Gr-IJP electrodes.

A long-term goal of our research is to develop electrochemical sensors and immunosensors to measure biomarkers of BRD in noninvasively collected EBC biospecimens. pH is one of the targeted biomarkers. During our pH sensor development work, it became apparent that multiple physiological and environmental variables can significantly influence the pH of bovine EBC. We present two examples here. Figure 9 presents summary pH data for EBC biospecimens from multiple healthy and ill calves. All pH values were measured using PANI/PA/Gr-IJP electrodes. Multiple IJP electrodes were calibrated in freshly prepared BR buffer solutions immediately before measuring the biospecimen pH, as described previously. Figure 9a presents time dependent pH data from the same three healthy calves. The data reveals a consistent finding that the EBC pH from the healthy calves depends on their age, trending more alkaline starting at 20 days. There is good agreement in the pH values determined using the PANI-modified electrochemical sensors (blue markers) and the micro glass pH electrode (black markers). For these three calves, the pH ranged from 5.4 to 6.9 over the period. The diets, environmental conditions, and time of the year when the EBC was collected were the same for all three animal subjects. Similar age-dependent trends were observed from most of the healthy calves in the study. The EBC collected during winter was ∼0.5 pH units more acidic than specimens collected during the summer. While we do not yet understand the biochemical origins, age is a variable influencing the EBC pH.

Figure 9b presents aggregated pH data for EBC collections from six healthy calves, all different ages from 1 to 9 weeks. Multiple collections from the same animals were analyzed. The collections were made during the summer and winer months, but otherwise, the diet and environmental conditions were the same for all the animals. The data reflect a total of 27 EBC measurements. Clearly, there is a large spread in the values from 5.3 to 7.2. Data for the 14 ill calves are also presented. These were all single EBC collections from the animals during the winter months. There is much less spread in the data as the pH values range from 5.4 to 6.4. It is expected that the EBC pH of the ill calves will be different from the healthy controls. Our preliminary results suggest that other variables need to be accounted for such as the time of year of the collection, age of the animal, and ambient conditions to fully test this hypothesis.

**Figure 10:**
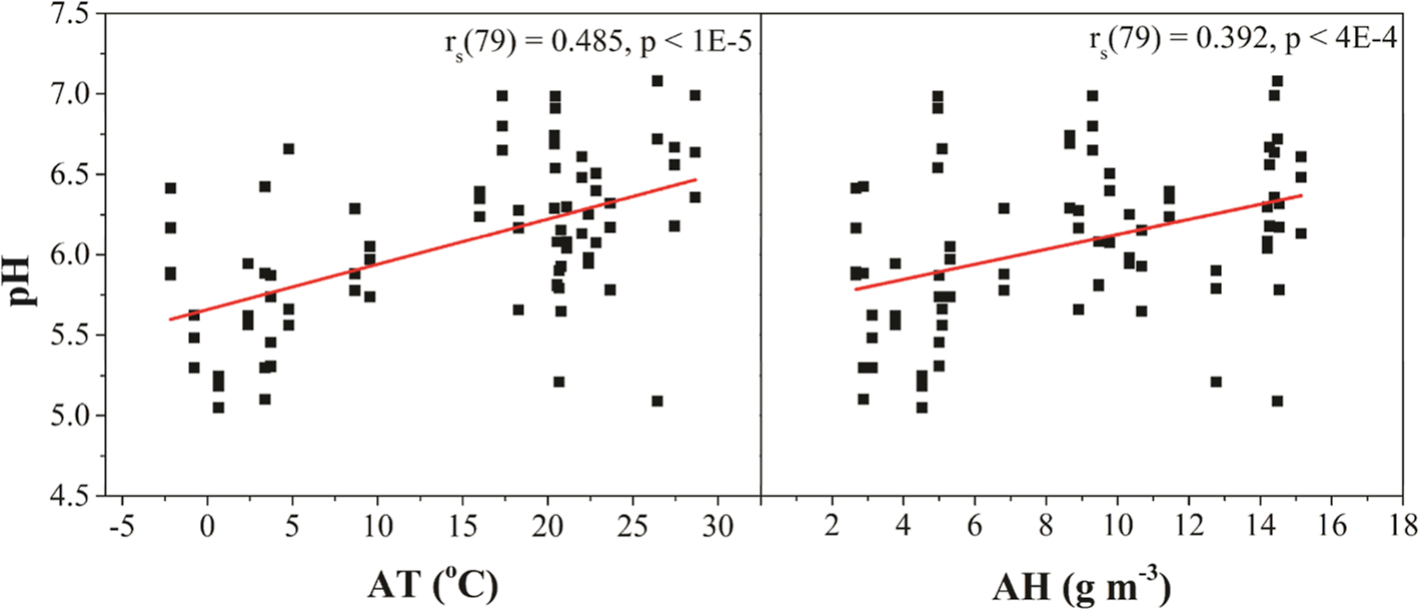
EBC pH measurement data for multiple healthy female calves (17 animals, 79 measurements) at different time points aggregated as a function of the outside (a) ambient temperature (AT) and (b) absolute humidity (AH) collected from each animal. The pH values were determined using the micro glass pH electrode. The Spearman coefficients (*r*_s_) for the pH vs AT and AH correlations were significant at *p* < 1 × 10^–5^ and *p* < 4 × 10^–4^, respectively.

The influence of the ambient conditions on EBC pH is demonstrated in Figure 10. The data were analyzed for correlations between the EBC pH of the healthy calves and their age, estimated weight, respiration rate (RR), ambient temperature (AT, or time of year), absolute humidity (AH), relative humidity (RH), and EBC volume collected. The figure presents data for how the EBC pH from healthy calves correlates with the AT and the AH. Spearman coefficients (*r*_s_) for correlations between all variables tested are shown in Table 3. The ambient conditions data were sourced from the MSU Enviroweather database (East Lansing, MI field station) at the time EBC was collected from the animals. The EBC pH trends more alkaline with increasing AT and AH (spring and summer versus fall and winter collection)^30^ and with volume collected. No significant correlation was found between EBC pH and RR or calf weight. Greater volume is collected from calves as they age and grow. The volume is dependent on lung capacity and the level of stress they experience during the EBC collection, i.e., higher RR. Although EBC pH and volume were significantly correlated, it is likely that this relationship originates from their mutual dependence on AT and AH. Importantly, the data reveals that there are several variables that can significantly influence the pH of bovine EBC.

**Table 3:** Spearman Coefficients for Correlations between Ambient Conditions or Calf Physiology and the EBC pH (Left Column) and EBC Volume (Right Column)^a^

parameter	EBC pH	EBC volume
ambient temperature	0.485, *p* ≤ 1 × 10^–5^*	0.696, *p* ≤ 1 × 10^–5†^
absolute humidity	0.392, *p* ≤ 4 × 10^–4^*	0.605, *p* ≤ 1 × 10^–5†^
relative humidity	–0.370, *p* ≤ 8 × 10^–4^*	–0.440, *p* ≤ 8 × 10^–5†^
respiration rate	0.328, n.s.^‡^	0.359, *p* ≤ 0.04^‡^
weight	–0.161, n.s.^‡^	0.392, *p* ≤ 0.02^‡^
EBC volume	0.450, *p* ≤ 5 × 10^–5†^	

a Results are reported as “*r*_s_, two-tailed *p*-value” for EBC pH measurements collected from multiple healthy animals. Correlations are presented for EBC collected from calves over a year (17 animals, either 79* or 75^†^ measurements) or between May–September (6 animals, 33 measurements^‡^). pH values were measured with the commercial micro glass pH electrode. n.s.: not significant.

**Figure 11:**
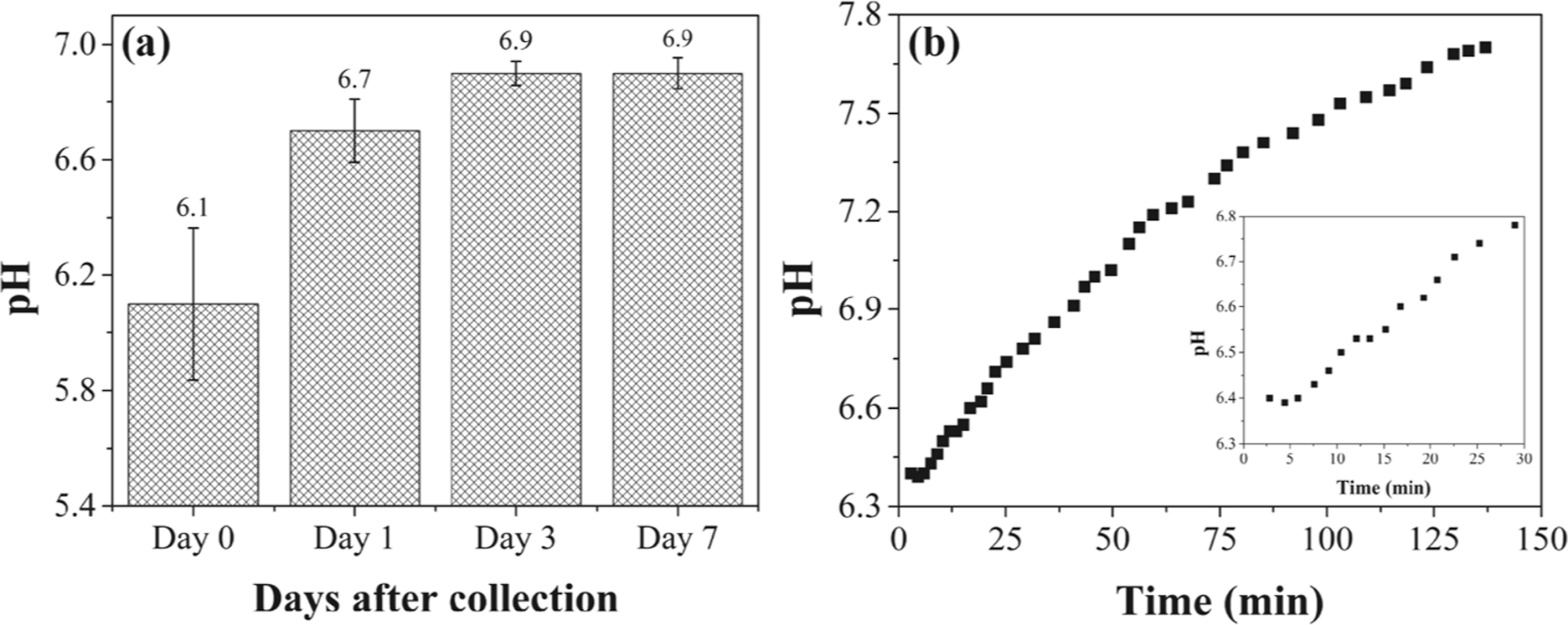
(a) pH measurement data for EBC biospecimens from four healthy calves before and after multiple freeze–thaw cycles over a 7 day period and (b) the time dependence of human EBC pH change during exposure to the ambient atmosphere. The inset shows the human EBC pH values between 2 and 30 min. The pH values were determined using the micro glass pH electrode. Data are presented as mean ± std. dev. for *N* = 4 EBC biospecimens from healthy animals.

Collected EBC, bovine or human, should be immediately analyzed or be stored at −80 °C until analysis. In our case, bovine EBC biospecimens were collected in the field, stored immediately on dry ice at −80 °C for about 1 h during the transport back to the laboratory, and promptly analyzed. The cold storage temperature is needed to reduce the rate of any chemical reactions that would alter the biochemical composition of the biospecimens and change the pH. Furthermore, it has been reported that repetitive freezing–thawing cycles of a biospecimens must be avoided, since this procedure results in loss of unstable chemical compounds.^60^ We performed pH measurements of the same four calf EBC biospecimens over three freeze–thaw cycles. To prevent the sublimation of volatile acids, the biospecimens were thawed while capped, shaken, and then uncapped for pH measurements. The results are presented in Figure 11a. The figure shows EBC pH data for four calves measured after multiple freeze–thaw cycles over a 7 day period. Immediately after processing and the initial pH readings (day 0), bovine EBC was stored at −80 °C for 24 h, thawed, then the pH rerecorded. This sequence was repeated after 3 and 7 days. Statistically significant increases, ∼0.5 pH units, were observed after 1 and 3 days of storage for all biospecimens. The pH stabilized in the 6.9 range. It should be noted that these pH values were obtained using the micro glass pH electrode and not the PANI/IJP electrochemical sensors. We attribute the increase in pH to the loss (exchange) of exhaled CO_2_ dissolved in the EBC with the ambient atmosphere.

One of the arguments against using EBC pH diagnostically is the fact that it is unstable due to carbon dioxide (CO_2_) equilibria. The dissolved CO_2_ concentration in solution, and therefore the pH, will depend on the atmospheric concentration of CO_2_. Our data are consistent with the latter. Figure 11b shows the time dependence of this CO_2_ exchange in a human EBC biospecimen after collection and processing. The pH increased about 1 unit over 130 min measurement period. Therefore, consistent sampling and processing methodology is critical for mitigating EBC pH fluctuations. In this study, the EBC pH was immediately measured after thawing to minimize ambient contamination with CO_2_. Therefore, it is supposed that our measurements were primarily influenced by the amount of exhaled CO_2_ from the animals with minimal contamination from the environment. CO_2_ (gas standardization) and inert gas purging has been recommended to achieve accurate and comparable pH measurements.^61^ Despite reproducibility advantages offered by these methods, some authors propose that immediate EBC pH analysis is more reflective of respiratory tract acidity and biochemical composition since it mitigates loss of EBC volume and volatile molecules (e.g., acetic acid, ammonia) during processing.^62,63^

### Comparison with Other PANI pH Sensors

The performance of the PANI/IJP electrode pH sensors were compared with other PANI-based pH sensors reported in the literature, as shown in Table 4. The PANI-electrodes reported in this work yielded near-Nernstian sensitivities, like those reported for sensors in other studies. The PANI/IJP electrodes developed herein exhibit short response times over a pH range of ∼7 units, especially the CNT-IJP electrodes. Additionally, the electrodes accurately measure pH of the noninvasively collected EBC. Overall, the PANI/IJP electrodes perform well as compared to other PANI-based electrodes reported in the literature.

**Table 4:** Comparison of Some Detection Performance Parameters of the PANI/IJP and PANI/SPC Sensors with Data Reported in the Literature for other PANI-Modified Sensors for pH Measurement^a^

sensor	sensitivity (mV pH^–1^)	time (s)	pH range	real sample	refs
(PANI-GA/GO)_3_/ITOLbL	–35.1	15	2–7	N.S.	(37)
PANI/rGO/ITO-PET	–62.3	50	2–8	sweat	(41)
GCE/GMC@PANI	–58.0	N.I.	2–11	urine and saliva	(43)
PANI/PS/SPC	–59.0	120	4–8	N.S.	(44)
PANI/CPE	–62.4	12.8	3–10	milk and apple	(45)
PANI/Au–Ti/PET	–60.3	N.I.	2–12	coke, coffee, water, orange juice	(46)
PANI/SPC	–52.3	180	2.3–9	EBC	this work
PANI/PA/CNT-IJP	–61.1	10	2.3–9	EBC	this work
PANI/PA/Gr-IJP	–51.6	30	2.3–9	EBC, saliva, wound swab	this work

a PANI: polyaniline; GA: gum arabic; GO: graphene oxide; ITO: indium tin oxide; LbL: layer by layer; rGO: reduced graphene oxide; PET: polyethylene terephthalate; GCE: glassy carbon electrode; GMC: graphitized mesoporous carbon; PS: polysaccharide; SPC: screen-printed carbon electrode; CPE: carbon paste electrode; Au: gold; Ti: titanium; CNT: carbon nanotube; Gr: graphene; IJP: inkjet-printed electrode; N.I.: not informed; N.S.: not studied.

## Discussion

One objective of this work was to compare the electrochemical performance of IJP carbon electrodes with the more conventional SPC version. The inkjet printing process offers some fabrication advantages over conventional screen printing including smaller dimensioned features, thinner layers of the active carbon electrode material, ease of fabricating multimaterial electrode structures, and ease of mass production. Inkjet printing has become an attractive alternative for the microfabrication of electrodes as they are easily produced by a mask-free and contact-less method. Other attributes include lower fabrication time and cost, lower ink waste (when drop-on-demand IJP is used), multimaterial deposition, and the possibility of using a variety of substrate materials. Compared to screen-printed electrodes, the electrode sizes can be smaller for IJP electrodes, reducing the sample volume needed for analysis.^33−36^ CNTs have been used as a functional material for the inkjet printing of working electrodes due to their electrical properties and chemical stability.^36^ For applications in complex samples, printing a hydrogel layer over the CNT electrode can inhibit or reduce electrode fouling.^33^ Overall, the detection figures of merit for the PANI-modified sensors and their use in pH measurements indicate that the IJP electrodes perform in a superior manner to the screen-printed counterparts. While the electrodes are designed for one-time use, the performance data presented herein show that the PANI/IJP electrodes exhibit little measurement hysteresis from sample to sample, even with minimal cleaning in between, less interference from other cations, and more rapid response stabilization times.

A second objective of this work was to design and test a field deployable device for reproducibly collecting EBC biospecimens from calves in the field. While there have been reports of bovine EBC collection,^64,65^ there is no commercial device available and no standard method for this collection. Our apparatus consists of a commercial animal anesthesia mask joined with a commercial RTube collection device. Using it, we were able to reproducibly collect EBC biospecimens in the field and transport them back to the laboratory for analysis. EBC collection from cattle is more challenging than collection from humans, as has been noted by Haddadi and co-workers.^2^ Cattle EBC is collected outdoors in the field and can be contaminated by chemicals in the environment. On the other hand, human EBC is collected in the indoor conditions of a medical facility. Furthermore, cattle rumination is a natural behavioral process involving regurgitation of previously consumed feed and masticating it a second time.^66^ The burp, which consists of various volatile organic compounds, will therefore mix with EBC and yield a biochemical composition that could differ from that of the ALF from which the EBC is generated. At birth, calves have small, undeveloped rumen that cannot digest solid foods. The rumen gradually develops with age through about 12–16 weeks old. During this process, calves transition from consuming milk or milk replacer to starchy foods.^67^ This differential diet and increased rumination over time could make it difficult to attribute pH or measurement of other biochemical species in EBC to the pathophysiology in the respiratory tract. Rumination in cattle typically occurs at night or during periods of extended rest.^67^ To lessen the effects of rumination on the EBC pH in this work, biospecimens were collected from 1 to 2 pm each day in between morning and evening feedings.^64^ Overall, the results demonstrate that the EBC collection procedure reproducibly provides 0.5–2 mL of EBC (6 min collection period) from healthy 1 to 9 week-old calves and that the biospecimens can be transported back to the laboratory and analyzed in a way that minimizes any biochemical degradation.

A third objective was to establish the range of EBC pH values in healthy calves and to learn what physiological and environmental factors affect the EBC pH using both the PANI-modified electrochemical sensors and the micro glass pH electrode. The results indicate that EBC pH in healthy calves ranges from 5.5 to 6.9 over 7 to 64 days of age with a distinct alkaline shift starting around 20 days of age. The values for all healthy calves studied at their different age points varied over a wide range from 5.3 to 7.2. While these calves differed in age, other variables such as diet, living environment, and ambient conditions were similar. This range of pH is larger than the pH range for the ill calves of 5.4–6.4. The diet and living environment for these animals were different from the healthy animals. Furthermore, two variables found to affect the EBC pH in healthy animals besides age are the AT and AH. Published research has associated these environmental variables with the prevalence and severity of respiratory tract infections.^68^ Other authors have documented an effect of meteorological fronts (weather conditions) on human EBC pH.^61^ In this work, we found the EBC pH tends to be more alkaline at higher AT and AH values. It is possible that the EBC pH is more alkaline during the summer months because respired air is warmer. CO_2_ is less soluble in the warm respiratory droplets and therefore the concentration available to react with water to form carbonic acid is lower. Conversely, cold air respired by the animals during winter months will have more solubilized CO_2_ in the respiratory droplets and therefore more carbonic acid formation and a more acidic EBC. An alternative explanation might be increased respiratory inflammation in response to cold, dry air. Prior published research has shown that cold temperatures increase the number of granulocytes and macrophages in the BAL of healthy human subjects,^69^ and inhaling dry air increases epithelial damage and inflammatory cell number in guinea pig trachea.^70,71^ Changes in these physiological markers indicate increased lung inflammation under cold or dry conditions. EBC is known to be acidified during acute lung inflammation, as indicated by elevation of inflammation biomarkers (e.g., interleukin-8, eosinophilia, neutrophilia).^26,72^ Identifying the biochemical origins of EBC pH variation with AH and AT is beyond the scope of this work.

## Conclusions

PANI was electrodeposited on PA/CNT-IJP and PA/Gr-IJP electrodes to make small, flexible pH sensors. A commercial SPC electrode was similarly modified for comparison. In this work, we tested these PANI-modified nanocarbon electrodes for their (i) analytical figures of merit toward pH sensing in standard buffer solutions and (ii) performance toward measuring the pH of several biospecimen types with validation using a commercial micro glass pH electrode. A focus of the work was on the collection and measurement measuring the pH of EBC biospecimens collected from healthy and ill calves suspected of suffering from BRD. Attention was paid to understanding how different variables (age, respiration rate, and ambient conditions) affect the bovine EBC pH. The homemade collection device worked well in the field and yielded 0.5–2 mL of EBC volume for 5–6 min of normal breathing.

The PANI/PA/CNT-IJP electrodes were the best performing overall, exhibiting a nominal slope of −61 mV pH^–1^, a response time ≤10 s, a response variability ≤7.8% RSD, and a linear dynamic range (*R*^2^ = 0.9986) from pH 2 to 8. The PANI/PA-Gr-IJP electrodes exhibited a slope of −52 mV pH^–1^, a response time ≤30 s, a response variability of ≤1.4% RSD, and a linear dynamic range (*R*^2^ = 0.9993) from pH 2 to 9. Both PANI-modified IJP electrode types were resistant to hysteresis effects and the PANI/PA/Gr-IJP electrode was minimally affected by possible interfering cations commonly found in EBC. The PANI/PA/Gr-IJP electrodes effectively measured the pH of human EBC, bovine EBC, human saliva, and canine wound swab biospecimens with validation using a mini-glass pH electrode. There was no significant difference between the pH of bovine EBC collected from healthy and suspected ill animals as measured with the PANI/PA/Gr-IJP electrodes. There are several variables that influence bovine EBC pH. First, the EBC pH of healthy calves depended on animal age. The EBC was acidified when the calves were about 20 days old, followed by a distinct alkaline shift with age. Second, higher ambient temperature and absolute humidity are associated with more alkaline EBC pH values. Our results confirm that bovine EBC pH can be measured using the PANI-modified sensors but there are multiple factors that influence EBC in healthy animals and these variables need to be better understood and accounted for if EBC pH is to be used as a predictive biomarker of BRD status.

## List of Abbreviations

AT: ambient temperature
AH: absolute humidity
BR: Britton Robinson
CNT-IJP: carbon nanotube-inkjet printed
EBC: exhaled breath condensate
Gr-IJP: nanographene-inkjet printed
PA: polyacrylamide
PANI: polyaniline
RH: relative humidity
RR: respiration rate
SPC: screen printed carbon
